# Effect of Chromosomal Instability on the Mutation-Selection Balance in Unicellular Populations

**DOI:** 10.1371/journal.pone.0026513

**Published:** 2012-05-23

**Authors:** Eran Itan, Emmanuel Tannenbaum

**Affiliations:** Department of Chemistry, Ben-Gurion University of the Negev, Be'er-Sheva, Israel; National Cancer Institute, United States of America

## Abstract

This paper develops a mathematical model describing the evolutionary dynamics of a unicellular, asexually replicating population exhibiting chromosomal instability. Chromosomal instability is a form of genetic instability characterized by the gain or loss of entire chromosomes during cell division. We assume that the cellular genome is divided into several homologous groups of chromosomes, and that a single functional chromosome per homologous group is required for the cell to have the wild-type fitness. If the fitness is unaffected by the total number of chromosomes in the cell, our model is analytically solvable, and yields a mean fitness at mutation-selection balance that is identical to the mean fitness when there is no chromosomal instability. If this assumption is relaxed and the total number of chromosomes in the cell is not allowed to increase without bound, then chromosomal instability leads to a reduction in mean fitness. The results of this paper provide a useful baseline that can inform both future theoretial and experimental studies of chromosomal instability.

## Introduction

Living systems have evolved a range of mechanisms to ensure the accurate transmission of their genetic information from one generation to the next [1]. When one or more of these mechanisms break down, the result is generally significantly increased mutation rates and variability in the distribution of genotypes in a population. Very often, the variability in the distribution of genotypes manifests itself through organizational changes to the genome itself, as opposed to point-mutations along the DNA gene sequences. When this happens, the genomes are said to exhibit *genetic instability* [1], [2], [3].

Genetic instability is a hallmark feature of cancer cells, and generally comes in one of two different forms: (1) Microsatellite INstability, or MIN, tumors, are characterized by elevated point-mutation rates and the accumulation of sequences of DNA in their chromosomes [1], [2], [3], [4]. (2) Chromosomal INstability, or CIN, tumors, are characterized by a breakdown in chromosomal segregation mechanisms during cell division, leading to the gain or loss of entire chromosomes [1], [2], [3], [4]. Such cells may no longer be diploid, with different homologous groups being characterized by different copy numbers, a phenomenon known as *aneuploidy* [1], [2], [3], [4]. In addition to the gain or loss of entire chromosomes, CIN is also characterized by the fusion of parts of chromosomes to one another, leading to more complicated genome-wide re-arrangements [1], [2], [3], [4].

The CIN instability is by far more prevalent than the MIN instability [1], [4]. Given the centrality of genetic instability to the progression of cancer, an understanding of the role of CIN on the evolutionary dynamics of a cellular population is important for developing accurate models of cancer progression, and for possibly assessing the efficacy of various treatment strategies.

## Materials and Methods

To model the evolutionary dynamics associated with the CIN instability, we consider a unicellular population of asexually reproducing organisms. We also assume that the organismal genome is organized as follows: We let 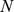 denote the total number of homologous groups in the genome. Labelling the homologous groups 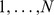, we let 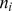 denote the number of chromosomes in homologous group 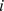 (by a homologous group, we mean a set of chromosomes that contain the same sequence of genes, though corresponding genes on different chromosomes within a group may differ from one another due to mutations).

We assume that each chromosome is a double-stranded DNA molecule, and that, when each chromosome replicates, the two strands unwind, and each strand serves as the template for the synthesis of the corresponding daughter strand (a process known as *semiconservative* replication).

During normal cell division, the two daughter chromosomes of a given parent chromosome segregate into separate daughter cells. However, with chromosomal instability, it is possible that two daughter chromosomes will segregate into the identical daughter cell. We let 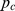 denote the probability that the two daughter chromosomes of a given parent segregate into separate daughter cells, so that 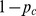 is the probability that they co-segregate into the same daughter cell.

We assume that DNA replication is not error-free. Thus, we let 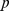 denote the probability that a given template strand of a given chromosome produces a daughter chromosome that is identical to the parent.

Following the single-fitness-peak approximation that is commonly used as a starting point in quasispecies and population genetics models, we assume that for each homologous group there is a wild-type chromosome for which that chromosome is functional. Any mutation to the wild-type is assumed to render the chromosome non-functional.

If the chromosomes are taken to be sufficiently long, then we may make an approximation known as the *neglect of backmutations*, which states that a template strand that differs from the wild-type will produce a daughter that differs from the wild-type with probability 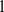. The basis for this assumption is that, for a sufficiently long chromosome, any new mutations will likely occur in a previously unmutated portion of the genome, so that the wild-type cannot be restored.

Finally, we assume that the first-order growth rate constant, or fitness, of a genome is determined by the number of functional chromosomes in each homologous group, as well as by the number of non-functional chromosomes in each homologous group. Thus, if 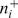 denotes the number of functional chromosomes in homologous group 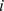, and 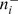 denotes the number of non-functional chromosomes, then the fitness is given by 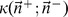, where 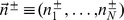.

When a given parent cell reproduces, it produces two daughters. Let us label these daughters as a “left” daughter cell and “right” daughter cell. Given a left daughter cell with genome characterized by 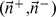, we define parameters 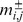, where 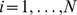, 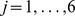 for 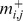 and 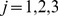 for 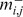, as follows:

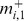
 denotes the number of functional chromosomes in homologous group 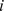 of the parent cell that do not exhibit instability, and that send a functional daughter into the left daughter cell. The probability that a given functional chromosome produces daughters that segregate in this manner is 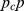.

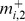
 denotes the number of functional chromosomes in homologous group 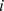 of the parent cell that do not exhibit instability, and that send a non-functional daughter into the left daughter cell. The probability that a given functional chromosome produces daughters that segregate in this manner is 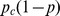.

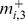
 denotes the number of functional chromosomes in homologous group 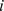 of the parent cell that exhibit instability, and send two functional daughters into the left daughter cell. The probability that a given functional chromosome produces daughters that segregate in this manner is 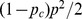.

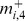
 denotes the number of functional chromosomes in homologous group 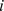 of the parent cell that exhibit instability, and send one functional and one non-functional daughter into the left daughter cell. The probability that a given functional chromosome produces daughters that segregate in this manner is 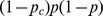.

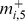
 denotes the number of functional chromosomes in homologous group 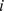 of the parent cell that exhibit instability, and send two non-functional daughters into the left daughter cell. The probability that a given functional chromosome produces daughters that segregate in this manner is 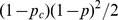.

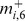
 denotes the remainder of the functional chromosomes in homologous group 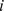 of the parent cell. These chromosomes must exhibit instability and send the daughters into the right daughter cell. The probability that a given functional chromosome produces daughters that segregate in this manner is 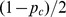.

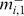
 denotes the number of non-functional chromosomes in homologous group 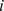 of the parent cell that do not exhibit instability. The probability that a given non-functional chromosome produces daughters that segregate in this manner is 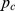, and such a chromosome will produce one non-functional daughter that segregates into the left daughter cell, and one non-functional daughter that segregates into the right daughter cell.

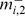
 denotes the number of non-functional chromosomes in homologous group 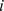 of the parent cell that exhibit instability, and send the daughters into the left daughter cell. The probability that a given non-functional chromosome produces daughters that segregate in this manner is 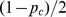, and such a chromosome will produce two non-functional daughters that segregate into the left daughter cell.

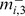
 denotes the number of non-functional chromosomes in homologous group 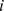 of the parent cell that exhibit instability, and send the daughters into the right daughter cell. The probability that a given non-functional chromosome produces daughters that segregate in this manner is 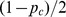, and such a chromosome will produce two non-functional daughters that segregate into the right daughter cell.

Now, for 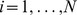, let 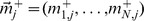, where 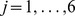, and 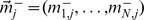, where 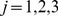. If 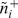, 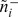 denote the number of functional and non-functional chromosomes in homologous group 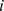, respectively, then we have,
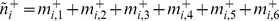


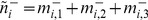
(1)For the left daughter cell, we also have,
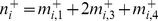


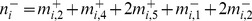
(2)These two equations imply that 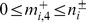, and so we have that 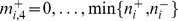. We then have that 
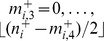
, 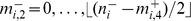, 
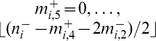
, 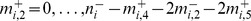, 
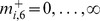
, 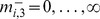.

Taking into account the transition probabilities listed above, as well as degeneracies in the ways we can choose the segregation patterns of the chromosomes consistent with given values of 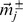, we obtain,
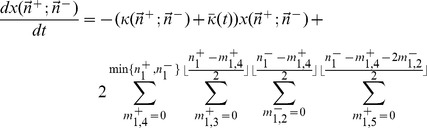


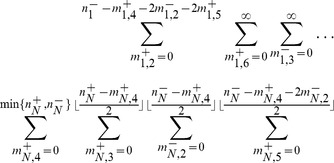


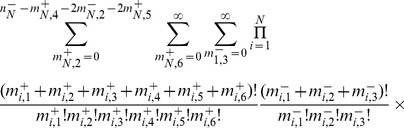


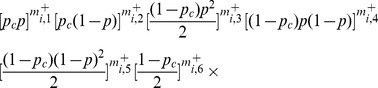


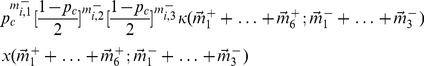
(3)where 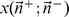 is the fraction of the population whose genome is characterized by 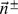, and 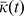 is the mean fitness of the population, or the average of all the first-order growth-rate constants weighted over the population distribution.

We assume that the fitness is either 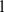 or 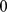. The fitness is taken to be 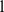 if each homologous group contains at least one functional chromosome, and if the total number of chromosomes does not exceed some value 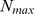. Otherwise, the fitness is taken to be 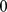.

If 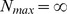, then the fitness only depends on the number of functional chromosomes in each homologous group, so that we may write 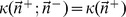. If we define 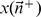 to be the total fraction of the population with 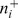 functional chromosomes in homologous group 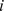, where 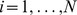, then by summing over all possible values of 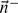, we obtain, after some manipulation,
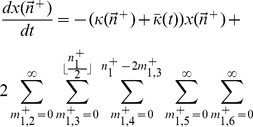


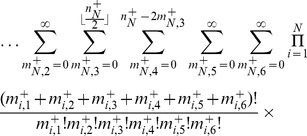


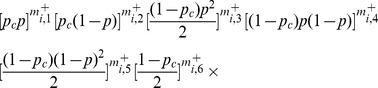


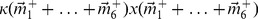
(4)


## Results and Discussion

We may analytically solve for the mutation-selection balance of Eq. (4). To do so, we define the quantity 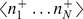 via,
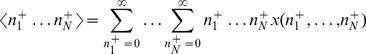
(5)Using Eq. (4), we can show, after some manipulation, that,
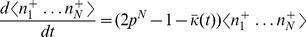
(6)Assuming that the system converges to a steady-state, and assuming that 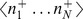 remains finite, we obtain that 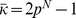. Interestingly, this suggests that the mean fitness at steady-state does not depend on the value of 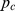.

**Figure 1:**
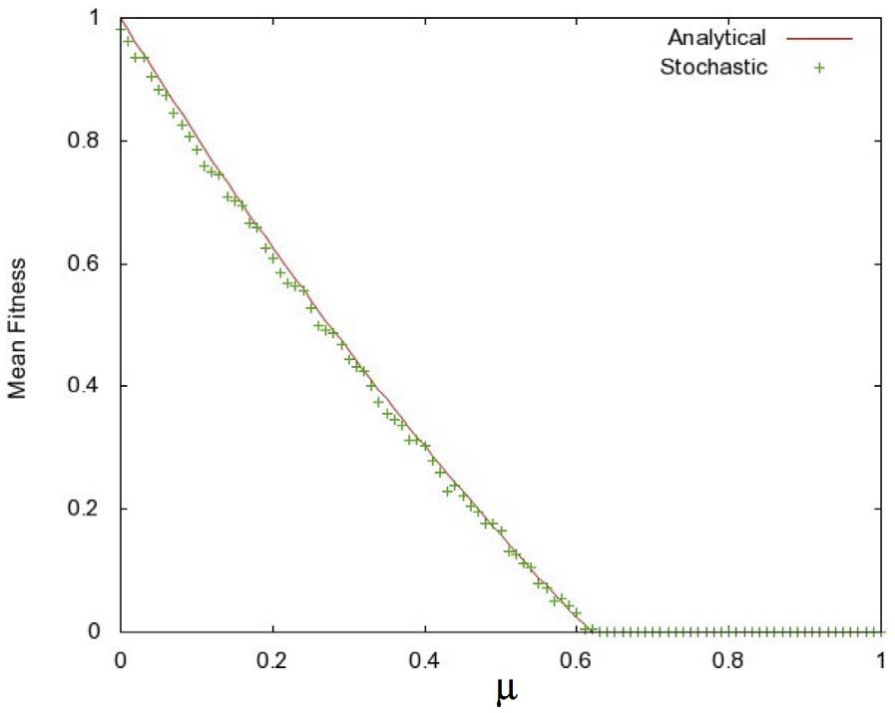
Comparison of the analytical expression for 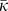 with the values obtained from stochastic simulations. We took 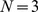, 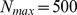, and 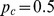. The stochastic simulation was run with a population size of 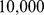, out to a time of 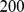 in time steps of 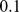, and with an initially clonal, wild-type population of haploids.

In Figure 1 we show a plot of the steady-state mean fitness, or 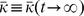, obtained from a stochastic simulation of reproducing organisms. Instead of plotting the mean fitness versus 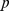, we plotted it versus 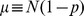. Note the good agreement between our theory and the simulation results.

Although we do not show plots for smaller values of 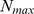, we do indeed find that, as 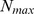 is decreased, the mean fitness drops, and may drop significantly below the 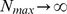 values.

Our result for the mean fitness hinges on the assumption that 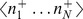 converges to a finite value. However, for 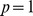, if this is the case, then it follows that 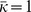. This implies, however, that 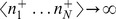 at mutation-selection balance for 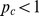. To see this, note that since cells with some 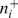 finite have a positive probability of producing a daughter without any chromosomes in homologous group 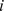, it follows that if 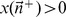 with some 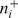 finite, then the subsequent dynamics will lead to the production of genomes with zero fitness, resulting in 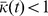, contradicting the fact that the population is at steady-state.

The way this issue is resolved is if 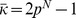 for 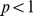, so that, by continuity, 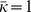 for 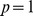. Also, 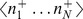 is finite for 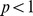, but infinite for 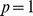. As a result, the convergence of 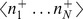 as a function of 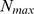 is not uniform, but rather must be increased as 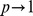. While we do not show plots of 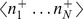, our stochastic simulation results suggest that the analysis above is indeed correct.

We developed a simpe mathematical model describing the evolutionary dynamics of a population exhibiting CIN. Our goal at this stage was not to model the dynamics of cancer progression, but rather to understand some basic features of the mutation-selection balance that is associated with CIN. We found that, under certain simplifying assumptions, that the mean fitness of the population did not depend on the extent of CIN. This is clearly an oversimplification, as we are neglecting additional features of CIN such as the fusion and deletion of parts of chromosomes, which can disrupt the normal sequence of genes and thereby affect the fitness of the population.

Another feature we are neglecting is the role of finite population size in our model, which can have a much stronger effect in the case of chromsomal instability. The reason for this is that, if a cell has a relatively large number of homologous pairs in the genome, and its initial ploidy level is low, then for a sufficiently high level of chromosomal instability it becomes likely that one daughter cell will not receive any chromosomes from some homologous group, while the other daughter cell will not receive any chromosomes from another homologous group. This will lead to a sharp initial decay in the population. If the population is sufficiently small, then this will lead to the extinction of the population. With an infinite population, the population growth rate does initially drop sharply. However, a small fraction of the cells will accumulate chromosomes in every homologous group. As the number of chromosomes in the homologous groups increases, these cells become shielded from the effects of chromosomal instability, and the population recovers as the mean fitness settles into a steady-state value. Thus, for future research we will need to develop stochastic models that explicitly take into account the finite size of the population.

Finally, we should emphasize that we are not the first to develop evolutionary dynamics models that incorporate CIN [2], [5], [6], [7]. Nevertheless, we believe that the model offered here provides additional perspective on the subject, and reveals new and interesting properties of replicator dynamics as applied to unicellular populations.

## References

[1] Attolini CSO, Michor F (2009). Evolutionary theory of cancer.. Ann New York Acad Sci.

[2] Brumer Y, Michor F, Shakhnovich EI (2006). Genetic instability and the quasispecies model.. J Theor Biol.

[3] Nowak MA, Komarova NL, Sengupta A, Jallepalli PV, Shih le-M (2002). The role of chromosomal instability in tumor initiation.. Proc Natl Acad Sci USA.

[4] Lengauer C, Kinzler KW, Vogelstein B (1998). Genetic instability in human cancers.. Nature (London).

[5] Michor F, Iwasa Y, Nowak MA (2004). Dynamics of cancer progression.. Nature Reviews Cancer.

[6] Michor F, Iwasa Y, Lengauer C, Nowak MA (2005). Dynamics of colorectal cancer.. Seminars in Cancer Biol.

[7] Nowak MA, Michor F, Iwasa Y (2006). Genetic instability and clonal expansion.. J Theor Biol.

